# The Care of Rheumatic Children

**Published:** 1913-01-18

**Authors:** F. J. Poynton


### THE CARE OF RHEUMATIC CHILDREN.

### Particularly of those with Heart Disease and Chorea.

By F. J. POYNTON, M.D., F.R.C.P. Lond.

[Read before a Meeting of the Council of the Invalid Children's Aid Association.]

Sir William Collins, Ladies and Gentlemen,—I must first thank you for your invitation and for an occasion which is of more importance to me than you can be aware. You have asked me to make some observations upon a subject to which I have devoted fifteen of the best years of my life, and which for the first time I have ventured to bring before an audience that is not a purely medical one. Every thinking person in such a position must needs feel a responsibility in these circumstances, because he is to some extent exempted from criticism.

Heart disease and chorea in childhood are disorders of the greatest importance, and possibly some of you hardly realise how frequently they occur as a result of rheumatism. It seems to me in our great cities rheumatism is almost as great a scourge as tuberculosis, and I look upon your invitation this evening as a welcome proof that there is a growing realisation of this fact.

The outstanding cause of heart disease and chorea is rheumatism, and the point which, with my colleague, Dr. Paine, I have been investigating for so many years has been the exciting cause of the rheumatism. I think we have shown without doubt that the cause is an infection, and further, I believe that we have demonstrated that the exciting agent is a diplococcus, which frequently gains access to the system through the inflamed tonsils. Our view has now been twelve years before the profession, and I think I may safely tell you that this rheumatism in childhood is now looked upon all over the world as an infective disease; although there are many who will not yet accept that we have demonstrated the only cause.

You must dismiss from your minds the idea that rheumatism is a disease only affecting the joints, and think of it now much as you think of tuberculosis—that is, as a disease affecting nearly every organ in the body. Yet I want to insist upon this point. When I say rheumatism is an *infective* disease I do not mean that it is an *infectious* disease. I only mean that the exciting cause is a microbe; so far as I can see, rheumatism does not spread from one child to another by direct infection, and we should be very wrong to look upon a rheumatic child much as a leper, an attitude of mind, in my opinion, too readily adopted in these days when with the public there is often some confusion between contagious, infectious, and infective.

Now the conception of this rheumatism as an infective process, together with the demonstration of the nature of the infection, brings us much nearer to the problem of grappling with its control. We know only too well that although in childhood rheumatism often attacks the joints and muscles, producing arthritis and so-called "growing pains," it attacks in particular the heart, producing heart disease, and the brain, producing chorea. Here, again, I must explain the part played by the infective agent in this disease, as I regard it. Those who study bacteriology are well acquainted with the fact that some microbes grow easily outside the body while others are very difficult to keep alive and require special foodstuffs. The interpretation is clear and of great practical importance; these microbes, though they may be the actual excitant of a disease in man, often require a special constitution or blood state to do their harm.

The rheumatic microbe outside the body is delicate and soon loses its character, and we notice that those who are prone to rheumatism are evidently also in some mysterious way peculiar in their constitution. It is a constitution, moreover, which is strongly hereditary. Then, again, the atmospheric conditions take an important part either in increasing the virulence of the microbe or in lowering the strength of the patient. Again, so far as we can see, damp, low-lying districts and damp houses and rooms are very likely to aggravate this rheumatism and make it more persistent. Poverty certainly helps to produce rheumatism, as you can well understand, for the poor child is more exposed and less well clothed and housed.

The importance of climatic conditions, however, must, I think, stand out clearly, for I have been struck with the repeated remarks of doctors from America, and from New York in particular, who have said that the rheumatism they see is a vastly different thing in childhood. The terrible cases we have here come as a revelation to them. I do not wish to trouble you with a number of statistics, but these figures will give you some idea of the nature of rheumatism in childhood. I took 500 consecutive cases coming to me and analysed the chief symptoms they showed on their first visit:—

248 complained of painful joints and muscles; 350 had heart disease; 245 had chorea; 137 tonsillitis more or less acute.

Rheumatism, then, must not be looked upon as a disease in which a microbe has only to enter the patient, like a penny slipped into a penny-in-the-slot machine, whereupon rheumatism will appear as does the box of matches. Unless the infection is virulent and beats down all resistance, it requires a peculiar constitution and other attending circumstances before it produces this rheumatism, and the process of infection is a complex one. Now much of the heart disease in early life persists through life—partly because rheumatism leaves scars which permanently damage the valvular mechanism, and partly because rheumatism tends to recur and attack the heart again and again. The early heart disease, then, is of immense importance, and we are met together here to consider among other points what we can do to prevent or minimise that evil.

The effect of rheumatism on the brain is somewhat different: rheumatism damages it frequently, but not as a rule permanently. Chorea, so frequent among the girls of our Council schools, becomes rare after puberty, and this is presumably due to some alteration in the brain with development which makes it more resistant. Nevertheless, during the school age chorea is a very serious illness, not only because it is so frequently associated with heart rheumatism, but also because it is so stubborn and so likely to recur and so very detrimental to the health and education. Of even more importance, however, is the fact that a child with chorea very frequently has also heart disease.

Now the symptoms of heart disease in children are often very few and easily overlooked, but the movements of chorea at once attract attention and so help us to detect heart disease by our knowledge of their frequent association.

There seems to me that a great deal can be done in attempting to diminish the severity of rheumatism. We cannot alter the climate and we cannot make the poor rich, but we can steadily, yet not aggressively, teach the parents.

The school inspection of children is a great step forward, if it is combined with common sense and a knowledge of possibilities and limitations. Much more, too, could be done for a child with rheumatism if the disease was more generally recognised.

It has been my rule for years to give the parents of the rheumatic a few simple instructions:—

1. A sore throat with pains in the limbs needs instant care.
2. Vague pains, called often "growing pains," should suggest the beginning of rheumatism.
3. Rheumatism is particularly likely to recur in autumn, winter, and spring, when there are sudden changes of temperature.
4. Heart disease is the great danger in rheumatism. This need not cause any pain, but often causes breathlessness, weakness, and anæmia.
5. St. Vitus's dance, or chorea, is almost always a sign of rheumatism. Nervousness, dropping things and alteration in disposition may be warnings of its approach.
6. Damp boots and clothes and damp rooms are highly dangerous to the rheumatic.
7. Heart disease requires great patience and much time for its treatment.

I think these simple directions do some good. The general public—as is only to be expected, for it had been until comparatively recent years the attitude also of the medical profession—is still inclined to look upon this rheumatism as an acute disease, at once prostrating the patient with pain, swollen joints, and fever. This conception is based upon the condition as it often shows itself in adults; but in childhood the onset may not only be acute and obvious, but also often very gradual and insidious. In this respect it recalls tuberculosis. I have no doubt that some of the almoners must get quite irritable with doctors who say "I suspect this child is tuberculous, but I am not sure." It is so also with rheumatism. I have watched a child for months, suspecting from such vague warnings as obscure pains, wasting, occasional fever, and slight disorderly movements, the occurrence of rheumatism, and then some event has at last determined an obvious attack. Here, then, we have a difficult problem in our subject, which we must face with patience while the disease is being more thoroughly worked out, and we must not hurry into alarmist measures. We cannot as yet make a child an invalid for every passing ache and pain, or keep him from school because he drops a plate or is troublesome.

I come now to a very definite indication for advance in treatment. It is this: Rheumatic heart disease requires a prolonged convalescence, although often enough there are no striking symptoms of illness. This brings two difficulties. The first is that this heart disease is so common that we cannot possibly treat it completely in our great hospitals.

We are obliged to recognise the functions of these hospitals. There are always urgent cases pressing to get in, and the teaching of the students must be wide in scope and varied in nature. If we filled the beds with chronic cases the acutely ill must suffer, and suitable training of medical students would be impossible. Yet there are few convalescent homes that are fitted for these patients, and I have been so disappointed with them that I rarely send a case of any decided heart damage to any ordinary home; and this, too, was the experience of the late Dr. Cheadle. It is not the fault of the homes, but these children want special care and watchfulness. For years I have been calling attention to the need of some special homes which should be first of all attached to the great children's hospitals. They could be started on a small scale, and I am quite sure, if we only persevere, some philanthropists will eventually come to our assistance. If there is anyone here to-day who by any influence can help forward the scheme I shall be glad; and I can assure everyone that the prolonged care of a child with a first attack of cardiac rheumatism, with or without chorea, will bring a great reward. Such homes, preferably inland, on a dry soil and in a sunny neighbourhood, would be built with few stairs and be gradually equipped with means for testing the improving strength of the heart.

The other difficulty is that the poor cannot realise this need for care because their children suffer no great pain, or perhaps they live up at the top of a house with steep stairs and care is impossible. They may even suspect their doctor of making undue fees and kindly visitors of being intrusive and interfering.

The influence of school upon chorea is a very difficult question, and one not to be rushed into hastily. Undoubtedly these highly nervous rheumatic children—often, I may add, bright and intelligent—frequently develop chorea which, I think, is actually determined by the strain of school work when they are in a rheumatic state. A child with commencing rheumatic heart disease is, we know, gravely injured by anything which tries the heart at that time, and rest is imperative. So, too, I think it is reasonable to suppose if the brain is exercised under the same circumstances—that is, when it is rheumatic—the disease will gain ground there. On the other hand, when the active disease has died down, the brain is so delicate an organ that signs of the illness may still remain, and then we may have the choice of a very unsatisfactory life at home or a comparatively disciplined one at school. We want more facts about this aspect of the question, and we shall no doubt soon get them. It is clear, however, that the special convalescent homes would help us much in completing the recovery from chorea as well as heart disease.

There is another problem before us, and that is the care of children with chronic heart disease. We shall always have these cases unless our medicinal measures for the treatment improve. We cannot at present prevent scars forming in the heart and damaging the valves, although we may do much to minimise the evil and strengthen the heart. Again, we have much to learn as to the capabilities of these children and to what extent ordinary school life is permissible. It is no use, in my opinion, to cast them adrift from school as a matter of routine; each case needs consideration. Special schools or rooms would be of much assistance, but the expense must be considered discreetly, for it is obviously not advisable to over-burden the healthy and weigh them down with enormous taxation by making hasty, half-thought-out schemes on great scales. A good deal can be done for these children by supervision, and I keep them year after year under observation, and should like to be able to send them away from time to time to suitable resting homes out of London.

There is nothing remarkable about their diet, and I have long ago abandoned the notion that meat is detrimental. Warm clothing is very essential. The condition of their throat and tonsils and teeth also requires attention, and much more care should be devoted to their occupation in life in the future.

This concludes my remarks. As sure as I stand here to-day, the time is coming when the care of the rheumatic child will meet with much more attention in this country.

